# Case Report: A rare case of complex Behçet’s disease complicated with acute tubular necrosis and IgA nephropathy, coexists with myelodysplastic syndrome, trisomy 8 and intestinal involvement

**DOI:** 10.3389/fimmu.2025.1618495

**Published:** 2025-09-15

**Authors:** Peipei Zhang, Wen Zhang, Hong Xia, Zhenliang Fan, Junfen Fan, Hongzhen Ma, Chunli Zhang, Shuyan Liu

**Affiliations:** ^1^ Department of Nephrology, the First Affiliated Hospital of Zhejiang Chinese Medical University (Zhejiang Provincial Hospital of Chinese Medicine), Hangzhou, Zhejiang, China; ^2^ Zhejiang Key Laboratory of Research and Translation for Kidney Deficiency-Stasis-Turbidity Disease, Zhejiang-Macau International Joint Laboratory of Integrated Traditional Chinese and Western Medicine for Nephrology and Immunology, Hangzhou, Zhejiang, China; ^3^ Department of Pathology, The First Affiliated Hospital of Zhejiang Chinese Medical University(Zhejiang Provincial Hospital of Chinese Medicine), Hangzhou, China; ^4^ Department of Hematology, The First Affiliated Hospital of Zhejiang Chinese Medical University(Zhejiang Provincial Hospital of Chinese Medicine), Hangzhou, China

**Keywords:** Behçet’s disease, acute tubular necrosis, IgA nephropathy, myelodysplastic syndrome, intestinal involvement

## Abstract

Behçet’s disease (BD) is a rare systemic disease in which small-vessel vasculitis impacts multiple bodily organs. It is typically marked by recurrent oral and genital ulcers, uveitis, and cutaneous lesions. However, peripheral vessels, cardiovascular structures, central nervous system, gastrointestinal tract, joints, lungs, or kidneys may be affected as well. Renal involvement, although uncommon, may manifest as proteinuria, hematuria, and varying degrees of renal insufficiency. Herein, we describe a 35-year-old man with longstanding BD, myelodysplastic syndrome (MDS), and trisomy 8. He presented with cutaneous erythema and gastrointestinal bleeding (requiring colonic resection), later developing acute renal failure. Features of both acute tubular necrosis (ATN) and IgA nephropathy appeared on subsequent biopsy. Following continuous renal replacement therapy and intravenous methylprednisolone treatment, there was gradual recovery of renal function. This scenario represents a rare and severe multisystem presentation of BD with complex comorbidities, attributing the observed kidney injury to combined insults as above. Given the persistent and multifaceted nature of BD, early recognition and targeted management of renal complications are essential to preserve functional capacity and improve patient outcomes.

## Introduction

Behçet’s disease (BD) is a multisystem inflammatory disorder characterized primarily by small-vessel vasculitis. Its prevalence is higher in regions along the ancient Silk Road (1). Skin, genitals, and gastrointestinal (GI) tract are most often affected, whereas an association with myelodysplastic syndrome (MDS) is relatively rare (2). Renal involvement in BD is also uncommon. According to past reports, amyloidosis is the prevailing pathology, followed by chronic glomerulonephritis and less frequently by renovascular disease (3). This account serves to document a rare and complex case of severe BD in the context MDS and trisomy 8—one that is complicated by biopsy confirmed acute tubular necrosis (ATN) and IgA nephropathy in the wake of intestinal necrosis.

## Case presentation

The patient was 35-year-old man with longstanding BD whose 3-week elevation of serum creatinine (SCr) prompted hospital admission. BD was diagnosed 18 years earlier based on recurrent oral ulcers, persistent high-grade fever, erythema nodosum, and a positive pathergy test. Prednisone (30 mg/day) and thalidomide were given at the time and seemed advantageous, conferring clinical benefit. Although prednisone tapering and discontinuation took place after 1 year of therapy, single doses were still warranted on occasion to manage intermittent bouts of low-grade fever and oral ulcers.

One year previously, the patient again experienced recurrent high fever (recorded maximum, 40.5°C), with relapse of oral ulcers, pharyngalgia, and headaches. Laboratory testing revealed pancytopenia (white blood cell [WBC] count, 0.7 × 10^9^/L; hemoglobin [Hgb], 46 g/L; platelet count, 47 × 10^9^/L) and remarkably high C-reactive protein (CRP, 91.88 mg/L). Empiric antimicrobial therapy and blood transfusion proved ineffective, but myelodysplastic syndrome (MDS) and trisomy 8 were diagnosed through fluorescence *in situ* hybridization and tumor mutational burden analyses of aspirated bone marrow.

Approximately 11 months prior, the patient claimed onset of hematochezia, reported as dark-red, jelly-like stools. Colonoscopy revealed severe ileocolonic changes likely stemming from BD. Treatment included intravenous (IV) methylprednisolone (80 mg/day) and immunoglobulin (10 g/day, 3 consecutive days), plus oral thalidomide (100 mg nightly). Unfortunately, the bleeding did not abate, instead culminating in massive GI hemorrhage and shock. The resultant fall in Hgb level to 59 g/L necessitated total colectomy and ileostomy on an emergency basis.

**Figure 1:**
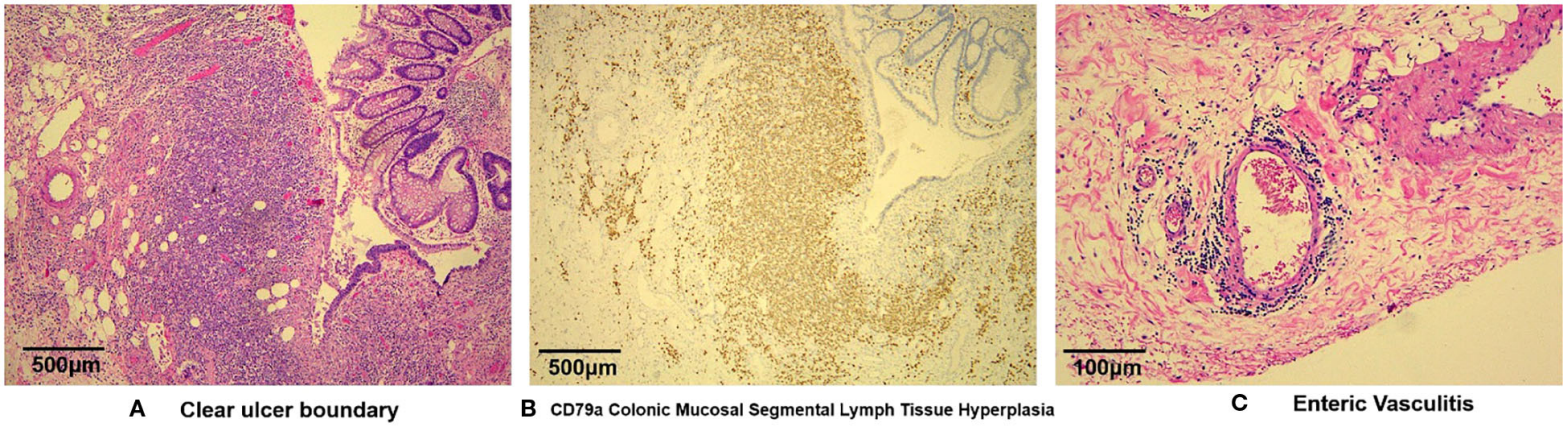
Microscopic views of vasculitis-induced intestinal necrosis: **(A)** full-thickness infiltration by chronic inflammatory cells at ulcer site, with necrosis and granulation tissue formation (H&E, 40X); **(B)** polyclonal proliferation of B lymphocytes differentiated from plasma cells (CD79a, 40X); and **(C)** mural infiltration of vessels by inflammatory cells and visible thrombosis, with luminal narrowing or obliteration—note partial arterial wall disruption and prominent histiocytic aggregates inside venous channels at ulcer base (H&E, 200X).

Histologic sections of resected colon confirmed vasculitis-associated intestinal necrosis. Full-thickness chronic inflammatory cell infiltrates were evident at ulcerative sites, in addition to necrosis and granulation tissue formation. Some blood vessels showed inflammatory mural infiltrates and visible thromboses, with luminal narrowing or complete obliteration (**Figure 1**).

During postoperative recovery, episodic seizures, with no abnormalities detected on brain MRI or CSF analysis, were successfully managed with oral sodium valproate. Ultimately, infliximab was administered at 250–300 mg (5 mg/kg) on Weeks 0, 2, and 6. This was followed by maintenance infusions at 8-week intervals. Thalidomide (100 mg/day) and cyclosporine (35 mg twice daily) were also added to the immunosuppressive regimen.

**Figure 2:**
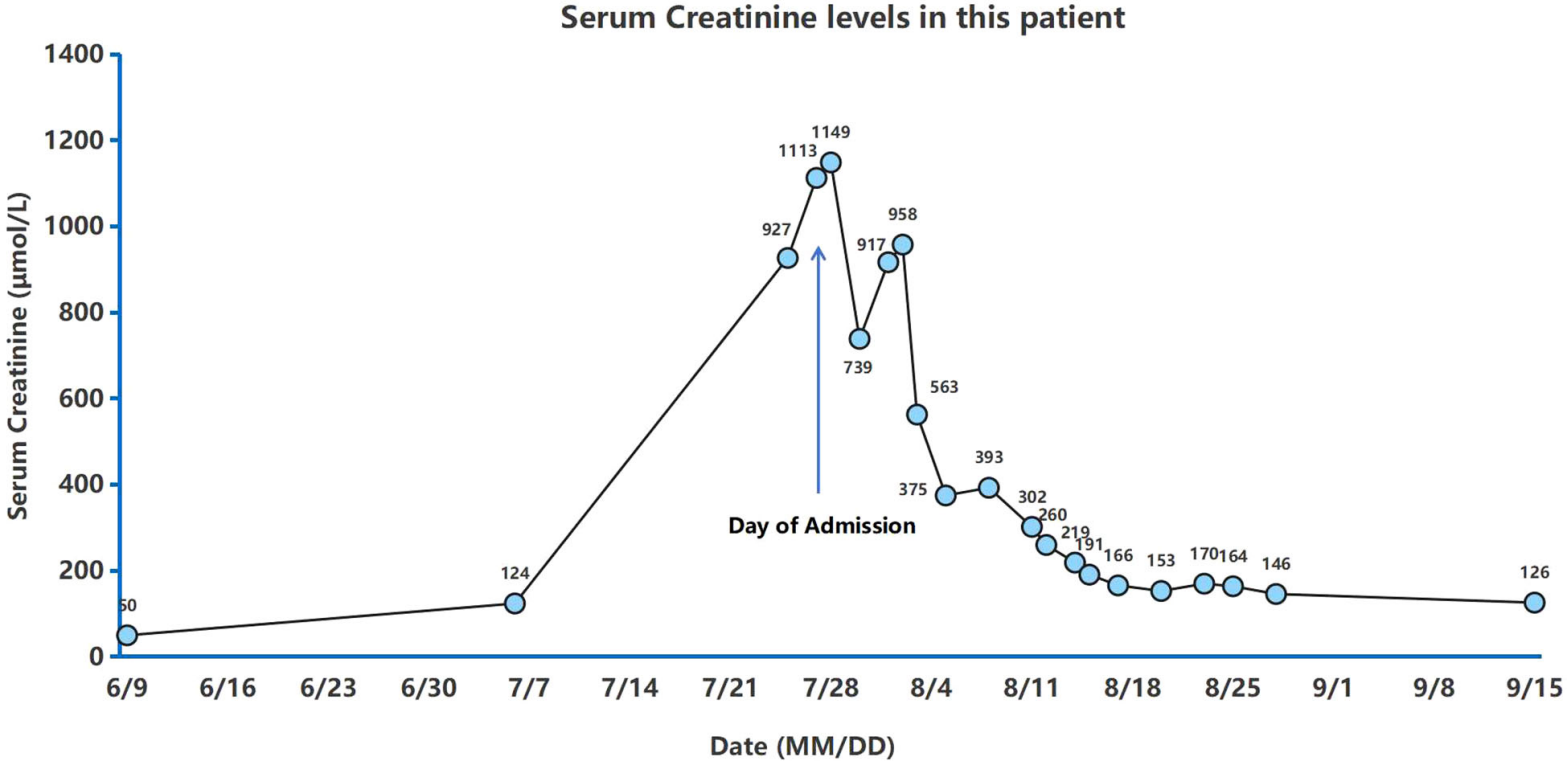
Dynamic changes in serum creatinine levels during patient’s clinical course.

**Figure 3:**
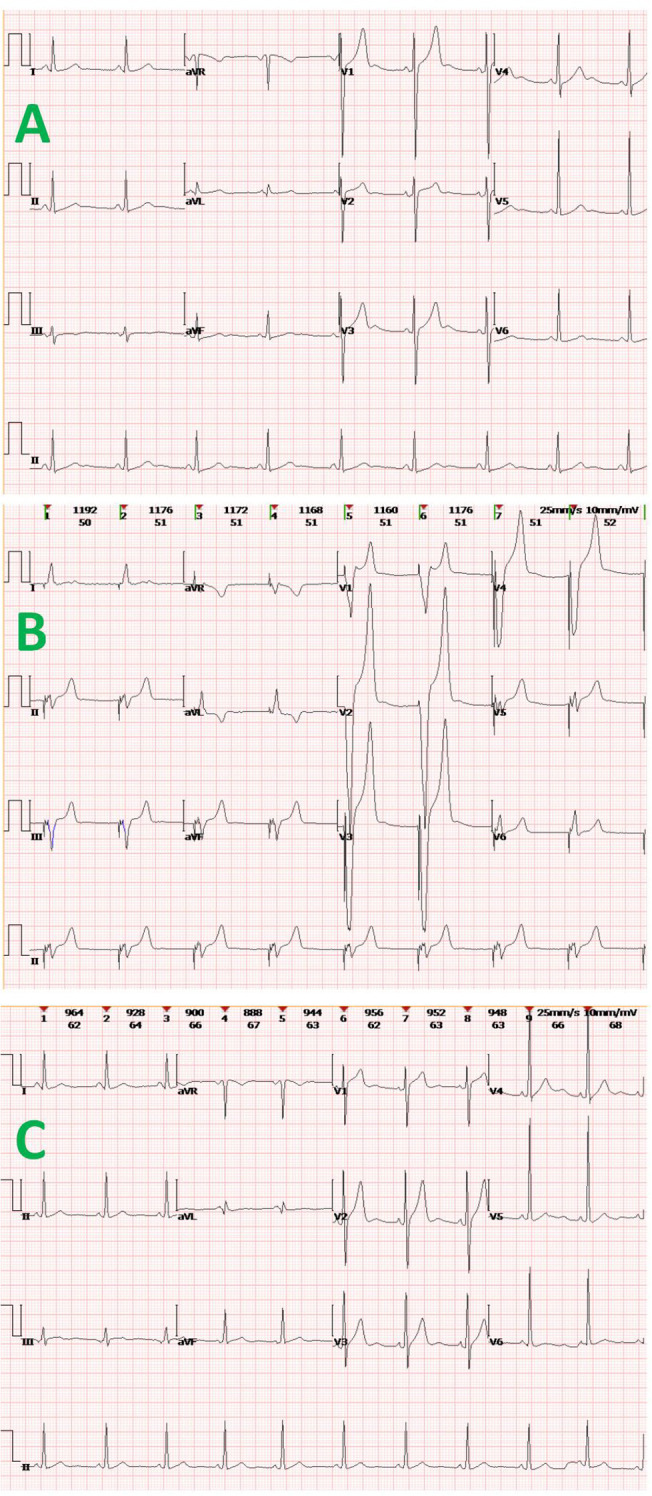
Electrocardiogram tracings at various clinical time points: **(A)** sinus bradycardia and mild QT-interval prolongation shown during acute renal failure; **(B)** post-implantation pacing in VVI mode, with broad/deformed QRS-T complexes following “spike-like” pacing signals; and **(C)** return to normal sinus rhythm.

**Figure 4:**
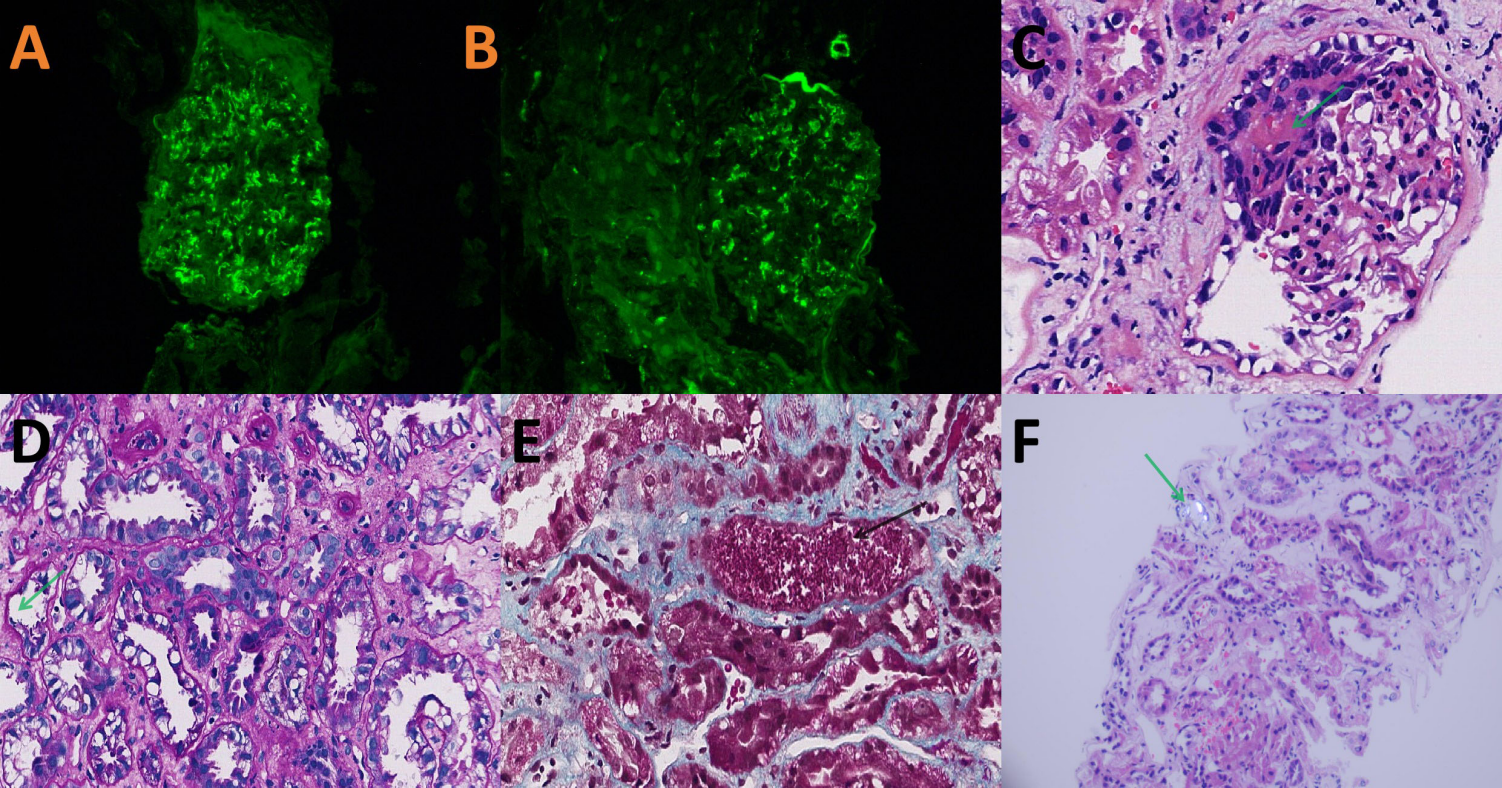
Renal biopsy findings: **(A, B)** acute tubulointerstitial lesions of IgA nephropathy clearly positive for IgA (+++) and C3 (++) by immunofluorescence microscopy, appearing as comma-shaped granular deposits in glomerular mesangial region and segmental capillary loops; **(C)** small cellular crescent containing fibrinoid exudate (arrow) (H&E, 200X); **(D)** vacuolar and granular degeneration of tubular epithelial cells, patchy tubular dilation, brush border loss, epithelial desquamation, and denuded basement membranes (green arrow) (PAS, 200X); **(E)** focal tubular necrosis and red blood cell casts (black arrow) (Masson, 200X); and **(F)** disc-shaped crystals within tubular lumen displaying white birefringence under polarized light (arrow) (H&E, 100X).

The patient had now presented with mild SCr elevation (up to 124 μmol/L) over a 3-week period. Urinary output was unfazed (~1,500 mL/day), but anorexia, nausea, and vomiting arose 3 days before admission, along with an upsurge in SCr (up to 1113 μmol/L) and hyperkalemia (6.1 mmol/L) (**Figure 2**). After concurrent initiation of volume resuscitation and potassium-lowering therapies, sinus bradycardia became problematic, registering a heart rate (HR) between 35 and 45 beats/min that required temporary transvenous pacemaker implantation (see corresponding electrocardiogram tracings of **Figure 3**). Once achieved, continuous renal replacement therapy (CRRT) began immediately, and renal biopsy was obtained when conditions permitted. In histologic sections, both IgA nephropathy and acute tubulointerstitial injury were identifiable. Chronic changes were also noted, including partial tubular necrosis. Moreover, birefringent disc-shaped crystals or basophilic deposits were discovered found within three tubular lumina under polarized light. The Oxford Classification of IgA nephropathy was M1 E0 S1 T0 C1 (**Figure 4**).

Renal replacement therapy continued, while administering IV methylprednisolone (80 mg/day) for 1 week. The patient gradually regained a normal heart rate (70 beats/min) in sinus rhythm, enabling pacemaker removal. After 3 weeks of treatment, he was discharged on prednisone tapered to 24 mg/day, with SCr at 166 μmol/L. Owing to a history of hepatitis B virus infection, antiviral therapy (tenofovir propofol fumarate, 25 mg/day) was additionally ongoing.

### Course of treatment

Upon admission, the patient underwent continuous blood purification therapy. Cyclosporine was discontinued, and intravenous methylprednisolone (80 mg/day) was administered for 1 week. The patient’s renal function gradually improved, with urine output increasing to 1,500-2,000 mL/day. Once stabilized, dialysis was terminated, and the dialysis catheter was removed.

### Prognosis and follow-up

At 1-year follow-up, a regimen of prednisone (7.5 mg, once daily), mycophenolate mofetil (250 mg, twice daily), and thalidomide (50 mg, once nightly) was in place. The most recent hematologic measures were as follows: WBC count, 1.9 × 10^9^/L; Hgb, 103 g/L; platelet count, 43 × 10^9^/L; and reticulocytes, 2.51%. CRP values had normalized, and proteinuria was absent on urinalysis, which did show 8.4 erythrocytes per high-power microscopic field. Uric acid (583 μmol/L), SCr (108 μmol/L), cystatin C (1.52 mg/L), and estimated glomerular filtration rate (eGFR, 79.15 mL/min/1.73 m²) were all within or near acceptable levels, reflecting adequacy of renal function.

## Discussion

### Renal involvement in patients with BD

BD is a chronic, relapsing, and multisystem autoimmune disorder with distinctive clinical features, including recurrent oral and genital ulcers, uveitis, and GI lesions (4). The diagnosis remains challenging due to lack of specific serologic markers, and disease severity may vary substantially among patients. Certain inflammatory cytokines, such as interleukin-6 (IL-6), have been proposed as auxiliary markers of disease activity (5). The sequence of events in our patient, marked by episodic oral ulcers and fever, diagnosis of MDS, catastrophic intestinal bleeding, seizure activity, acute renal failure, hyperkalemia, and obligatory temporary pacemaker implantation, was especially complex and overall proved quite daunting for clinical management.

The reported prevalence of renal involvement in BD varies widely across studies, ranging from 0-55% (3). These data may reflect differences in study populations, geographic regions, genetic predispositions, and diagnostic criteria, all of which influence accuracy and consistency of prevalence estimates. Abnormal urinalysis (i.e., proteinuria and microscopic hematuria) is the most frequent indicator of renal injury. Elevated SCr and blood urea nitrogen levels are reported in only a minority of cases (6), A subset of patients may also present with hypertension, often exhibiting malignant hypertension secondary to BD-related renal artery stenosis (7, 8). Histologic findings in renal biopsies from patients with BD have exposed an array of disease processes. Renal amyloidosis is most commonly reported, followed by chronic glomerulonephritis; and although rare, IgA nephropathy has been documented as well (9–11).

### Acute tubulointerstitial injury in relation to BD

Acute tubulointerstitial injury is an uncommon manifestation of BD. To date, this is the first reported case of BD to show concurrent ATN and IgA nephropathy in the setting of MDS. As a chronic, relapsing autoimmune disorder, BD typically calls for long-term immunosuppressive therapy using corticosteroids, conventional immunosuppressants, or biologic agents. However, a number of these are actually implicated in renal injury. Prolonged use of cyclosporine is associated with acute and chronic tubulointerstitial injury, including interstitial fibrosis (12). Biologic agents, such as adalimumab and infliximab, have also been linked to onset and progression of IgA nephropathy in some patients (13, 14). Rarely, BD-related acute kidney injury (AKI) may be pronounced, causing rapid elevations of SCr and life-threatening hyperkalemia. It is exceedingly rare for patients with BD to require both temporary cardiac pacing and CRRT, as we have detailed.

In our patient, calamitous GI bleeding created a surgical emergency. Still, intestinal resection and the functional impairment entailed are recognized risk factors for secondary oxalate nephropathy. Inflammatory bowel disease is known to increase intestinal binding of calcium to the surplus of unabsorbed fatty acids produced, thereby reducing fecal excretion of calcium oxalate. Hyperoxaluria is thus promoted through enhanced oxalate absorption. At this same time, decreased urinary excretion of citrate and magnesium—both inhibitors of calcium oxalate crystallization—further increases the risk of nephrolithiasis. Hence, intestinal surgery (ileostomy mostly) is an independent risk factor for urinary stone formation, due to chronic dehydration, bicarbonate loss, and resultant urine acidification (15).

Upon histologic examination, renal tubules in this patient harbored apparent oxalate deposits. Disc-shaped crystals showing white birefringence under polarized light were visible within the lumina, possibly contributing to tubular obstruction and exacerbating the acute kidney injury (**Figure 4**). In previously reported cases of BD-related renal compromise, glomerular pathology occasionally revealed crescent formation, which is associated with rapid disease progression and poor urinary outcomes (3, 16). However, glomerular involvement was mild in this instance, with renal injury largely confined to ATN and interstitial inflammation. Corticosteroid therapy is known to accelerate recovery from ATN. In addition to high-dose corticosteroids, CRRT likely helped to improve renal function.

### Intestinal involvement in patients with BD

Intestinal effects are inherent in BD, compelled by the systemic inflammatory nature of this disorder (17). Pertinent clinical symptoms commonly include abdominal pain, diarrhea, and hematochezia. In more severe cases, intestinal obstruction, perforation, or necrosis may emerge as complications. Management strategies for such involvement rely on both pharmacologic and surgical interventions. Immunosuppressive therapy, namely corticosteroids and calcineurin inhibitors, remain the cornerstone of medical treatment, aiming to control inflammation and alleviate GI symptoms (12). For disease refractory to conventional immunosuppressants, surgical remedies (ie, bowel resection and enterostomy) may be inevitable. Biologic agents targeting tumor necrosis factor-alpha (TNF-α), particularly infliximab and adalimumab, have demonstrated efficacy in those with intractable intestinal BD, facilitating symptom control and healing of extreme mucosal damage (18). Our patient’s uncontrolled GI bleeding and necrosis did not respond to therapy (corticosteroids, IV immunoglobulin, thalidomide, and cyclosporine), rendering surgical resection and ileostomy unavoidable. Infliximab administration in the aftermath finally brought clinical improvement and resolution of GI symptoms.

### Pathogenetic interplay between BD, MDS, and trisomy 8

The coexistence of BD and MDS represents a clinically significant entity characterized by overlapping pathophysiology. MDS comprises heterogeneous clonal hematopoietic stem cell disorders featuring ineffective hematopoiesis, peripheral cytopenia, and risk of leukemic transformation. Notably, epidemiologic studies report a 0.4-3.1% prevalence of MDS among BD patients (19–21), suggesting potential bidirectional pathogenetic links. Chronic immune dysregulation inherent to BD may drive clonal expansion of hematopoietic stem cells, while MDS-related immunosuppression can manifest as BD-like features including recurrent mucocutaneous ulcers. This clinical convergence complicates diagnosis and often delays appropriate intervention, particularly given the heightened gastrointestinal involvement observed in concurrent BD-MDS cases compared to isolated BD.

Central to this interplay may be trisomy 8, a cytogenetic aberration present in 7–9% of MDS patients with detectable chromosomal abnormalities (22, 23). Mechanistically, trisomy 8 drives overexpression of proinflammatory cytokines including transforming growth factor-β, IL-6, and interleukin-7 receptor in MDS (24), mirroring elevated IL-6, granulocyte colony-stimulating factor, and TNF-α levels in trisomy 8-positive BD patients. This shared cytokine dysregulation disrupts immune homeostasis, damages hematopoietic stem cells, and promotes both bone marrow failure and autoinflammatory manifestations. Critically, trisomy 8 constitutes a high-risk marker for BD-to-MDS progression (25) and correlates with severe gastrointestinal involvement. Systematic evidence confirms trisomy 8-positive patients exhibit significantly higher mortality (OR 11.74), gastrointestinal manifestations (76.1% vs. 17.2% in classical BD), and treatment-refractory inflammation, underscoring its distinct clinico-pathologic identity (26).

Therapeutic implications emerge from this unified pathogenic model: hematopoietic stem cell transplantation demonstrates disease-modifying potential in BD patients with concurrent trisomy 8-positive MDS (27), while conventional immunosuppressants and corticosteroids remain first-line options. Future research should clarify precise molecular pathways linking trisomy 8, cytokine networks, and hematopoietic clonality to optimize risk stratification and targeted therapies.

### Renal implications of MDS

MDS is a serious but rare clonal hematopoietic disorder. Assorted renal pathologies documented in conjunction with MDS include acute tubulointerstitial nephritis, ANCA-negative pauci-immune necrotizing and crescentic glomerulonephritis, membranous nephropathy, and IgA nephropathy (28–30). Among these, acute tubulointerstitial nephritis and membranous nephropathy are most common. Because the risk of renal biopsy is heightened in patients with MDS (due to cytopenia), biopsy proven cases are scarce. Past reports have thus failed to properly record simultaneous occurrences of BD, MDS, and renal pathology. The present account is the first to address coexistence of these three conditions, highlighting an unusual and complex clinical scenario.

## Conclusion

This report details a rare and severe case of BD leading to intestinal necrosis and subsequent acute renal injury (due to ATN, IgA nephropathy) in a patient with MDS and trisomy 8. It underscores the need for timely recognition and management of renal involvement in patients with BD, serving to preserve kidney function and improve clinical outcomes. Given the multisystem nature of BD, with potential overlap of hematologic and GI complications, a multidisciplinary approach to diagnosis and management is essential. Early detection, individualized treatment strategies, and continuous follow-up are critical for optimizing outcomes under such complex circumstances.

## Data Availability

The original contributions presented in the study are included in the article/**Supplementary Material**. Further inquiries can be directed to the corresponding author/s.
